# A cross-species framework to identify vocal learning abilities in mammals

**DOI:** 10.1098/rstb.2020.0394

**Published:** 2022-01-03

**Authors:** Andrea Ravignani, Maxime Garcia

**Affiliations:** ^1^ Comparative Bioacoustics Group, Max Planck Institute for Psycholinguistics, Wundtlaan 1, 6525 XD Nijmegen, The Netherlands; ^2^ Animal Behaviour, Department of Evolutionary Biology and Environmental Studies, University of Zurich, Winterthurerstrasse 190, Zurich 8051, Switzerland; ^3^ Center for the Interdisciplinary Study of Language Evolution, University of Zurich, Zurich 8032, Switzerland

**Keywords:** vocal production learning, acoustic allometry, phylogenies, outliers, vocal tract

## Abstract

Vocal production learning (VPL) is the experience-driven ability to produce novel vocal signals through imitation or modification of existing vocalizations. A parallel strand of research investigates acoustic allometry, namely how information about body size is conveyed by acoustic signals. Recently, we proposed that deviation from acoustic allometry principles as a result of sexual selection may have been an intermediate step towards the evolution of vocal learning abilities in mammals. Adopting a more hypothesis-neutral stance, here we perform phylogenetic regressions and other analyses further testing a potential link between VPL and being an allometric outlier. We find that multiple species belonging to VPL clades deviate from allometric scaling but in the opposite direction to that expected from size exaggeration mechanisms. In other words, our correlational approach finds an association between VPL and being an allometric outlier. However, the direction of this association, contra our original hypothesis, may indicate that VPL did not necessarily emerge via sexual selection for size exaggeration: VPL clades show higher vocalization frequencies than expected. In addition, our approach allows us to identify species with potential for VPL abilities: we hypothesize that those outliers from acoustic allometry lying above the regression line may be VPL species. Our results may help better understand the cross-species diversity, variability and aetiology of VPL, which among other things is a key underpinning of speech in our species.

This article is part of the theme issue ‘Voice modulation: from origin and mechanism to social impact (Part II)’.

## 1. Introduction

Understanding the vocal learning phenotype in humans and non-human animals has raised significant interest, most probably because this trait is a key prerequisite to human speech [1,2]. Cross-species investigations have been carried out to find the neurobiological [1] and social [3] underpinnings of this particular vocal ability. Why are some animals capable of vocal learning? Among the existing hypotheses (see [3–5]), we recently proposed that enhanced vocal flexibility associated with allometry-cheating vocalization may have paved the way to the emergence of vocal learning [4]. In brief, allometric scaling predicts that larger animals will produce sounds with lower frequencies [6–8]. In parallel, vocal learning species have so far been described as those that can imitate or learn sounds not belonging to their innate repertoire [9,10], although this is gradually changing in favour of a broader and more nuanced definition [11,12]. We wondered: is there an association between escaping from standard acoustic allometry constraints and being a vocal learner? Our preliminary inspection of an existing dataset featuring both vocal learners and non-vocal learners provided preliminary support to this hypothesis [4]. In particular, we highlighted a statistical relationship between being a vocal learner and being an outlier to standard acoustic allometry scaling. Here, we refine our preliminary analyses and ask: are the magnitude of deviation from an allometric regression and the direction of deviation predictors of vocal production learning (VPL) capacities?^1^

Typically, deviations from acoustic allometry principles highlight a form of dishonest signalling [6,7]. A signal is considered dishonest when an animal transmits information which does not fully reflect its physical or physiological condition, often for its own benefit (and with no implications of this process being intentional or conscious). A well-known example of this is the phenomenon of Batesian mimicry [13,14], where non-poisonous animals display warning signals which would normally be attributed to poisonous animals. Likewise, animal vocalizations which make an individual sound bigger or smaller than their actual body size are examples of dishonest signalling, characterized by deviations from acoustic allometry. How do we assess whether a species produces dishonest signals? One way to do this is to test for a linear relationship between animals' body size and the frequency of sounds they produce. Species that produce sound frequencies higher or lower than expected (based on an interspecific averaging) for their body size can be considered as being allometric outliers and engaging in a form of dishonest signalling. While in theory, dishonest vocal signalling can arise both from size-exaggeration and size-reduction mechanisms, documented cases have mainly provided evidence for the former (see [6] and references therein).^2^ In such cases, runaway selection can lead to permanent anatomical adaptations (e.g. in red deer [16] and in koalas [17]), which typically correlate with deviations from allometric scaling. Our preliminary analyses [4] also suggested similar deviations for VPL species, likely inherent to the increased vocal flexibility that characterizes VPL abilities. Without speculating on the role of evolutionary pressures, the focus of this paper thus consists of investigating whether species in clades with VPL abilities (VPL species thereafter) are ‘downward outliers’ (i.e. below an average interspecific acoustic allometry regression line, similar to what is observed for species with anatomical adaptations), upward outliers or simply outliers with no particular directional pattern.

## 2. Methods

### (a) Acoustic and body size data

Following our recent opinion piece [4], we used an already existing dataset (by Martin *et al*. [18]) from an acoustic allometry study compiling vocalization features across mammalian species. This dataset was selected as it includes as many mammalian species as possible for which acoustic measures were available, and also a good proportion of species from the four mammalian vocal learner clades (here defined as clades that include species with evidence of VPL abilities). The reported vocalization features correspond to the minimum and maximum dominant frequencies found across species' vocal repertoires, described by the authors as ‘the signal with the lowest, and the signal with the highest peak frequency’, respectively [18, p. 251]. It would have been ideal to carry out separate analyses relying specifically on the fundamental frequency (*f*_o_) and on formant frequency spacing (Δ*F*), following, for instance, an acoustic allometry study recently conducted by Charlton & Reby [6]. However, the dataset used in this recent study lacked identified vocal learner species for the purpose intended here, and we, therefore, decided to use the same dataset as for our original Opinion Piece [4]. Note that, because call types can be either tonal or involve nonlinear phenomena, we believe dominant frequency to provide a valid compromise to having separate analyses on *f*_o_ and Δ*F*. Indeed, in tonal signals, the dominant frequency generally matches *f*_o_ [19], while in chaotic and harsh calls, the dominant frequency more likely matches a formant frequency, since *f*_o_ is absent in such vocalizations. Body size has been typically approximated in acoustic allometry studies either by body length or by body mass, which have both shown to provide reliable results (e.g. [6,20]). In this study, the data used from Martin *et al*. [18] consist of the average body mass for each species.

### (b) Categorization of species as vocal learners

Although recent work calls for careful adjustments in categorizing vocal learning abilities along a multidimensional space [12], we applied a conservative approach in our choice to characterize species as vocal learners. We excluded, for instance, cases of vocal convergence, seen in Guinea baboons [11] and chimpanzees [21], although these could be considered part of a broad vocal learning contiguum [12]. We, therefore, classified as vocal learners the species that belong to the four non-human mammalian clades for which vocal learning has been shown in the form of VPL. We decided to extend the classification of ‘vocal learner’ to all species that are part of a clade for which VPL has been shown (as opposed to limiting our classification to species only) because experimental paradigms differed and provided evidence of VPL of variable strengths. These clades were, therefore, cetaceans, bats, elephants and non-otariid pinnipeds [12,22–25]. We excluded otariid pinnipeds given that this group does not seem capable of VPL [26,27] (despite otherwise similar general learning abilities in phocid and otariid pinnipeds). As in our previous work, we recognize this broad classification constitutes a limitation in our analysis, which should be improved as more and more species are shown capable of VPL (or at least appropriately tested for such abilities) and as the vocal learning contiguum framework is further expanded.

For robustness, additional analyses were also performed on two subsets of the data and are reported in the electronic supplementary material (see the section ‘Additional analyses’). In brief, the first subset included the whole dataset excluding Odontocetes, which produce sounds using non-laryngeal mechanisms and which could be unexpectedly high (i.e. echolocation clicks [28,29]). The second subset included all taxa for which there is no evidence for vocal learning plus the only 13 species of the full set for which there is clear evidence of vocal learning (i.e. in that case, we kept a strict definition of VPL without extending it to entire clades).

### (c) Phylogenetic analyses

We investigated the acoustic allometry relationships between body mass and acoustic parameters using phylogenetic generalized least square (PGLS) regressions using the ‘nlme’ package [30] of the R software [31]. Acoustic parameters considered based on Martin *et al*'s data [18] were the ‘minimum dominant frequency’ (MinDF) and ‘maximum dominant frequency’ (MaxDF), from which we additionally derived the ‘mean dominant frequency’ (MeanDF; the mean of MinDF and MaxDF) and the ‘range of the dominant frequency’ (RangeDF; defined as MaxDF–MinDF). Acoustic and body mass data for 164 species were retrieved from Martin *et al.* [18], and we used the mammalian phylogenetic tree from a recent study providing an up-to-date mammal phylogeny (http://doi.org/10.1016/j.ympev.2014.11.001; [32]). Note that, prior to running our analyses, we conducted a verification of frequency and body mass data and corrected obvious typos and omissions (see ‘List of modifications to the original dataset’ in electronic supplementary material). We refined the analysis carried out in our earlier work [4] by generating a consensus tree (using the ‘consensus.edges’ function from the ‘phytools’ R package [33]) from the multiPhylo object given as source data. More specifically, we generated one ‘average’ tree from a list of 1000 typologies of the full mammalian phylogeny, with edge (or branch) length computed as the mean edge length for each edge in the consensus tree. This consensus tree followed trimming of the multiPhylo source object to retain only the 164 species for which we had matching acoustic and body size data (which were all log-transformed prior to running the PGLS models). We believe this approach to be more relevant to the phylogenetic interpretations that we derive in the current study (e.g. by relying on single estimates from a PGLS model rather than on an average of estimates from multiple PGLS conducted on 1000 typologies). In order to choose the best among different evolutionary scenarios, following recent studies [6,19,34], we computed several PGLS models and selected the one with the best fit, i.e. the lowest Akaike information criterion corrected for sample size (AICc). These models were implemented using the ‘Ape’ package [35] and included a pure Brownian model of evolution (using the function ‘corBrownian’), a Brownian motion + Pagel's Lambda model (BM+*λ*; using the function ‘corPagel’), a Brownian motion + Grafen's *ρ* (BM+*ρ*; using the function ‘corGrafen’), and an Ornstein–Uhlenbeck (OU; using the function ‘corMartins’) model which can simulate a stabilizing selection (represented as an *α* parameter) to transform the tree. Note that due to model computation on our consensus tree, the stabilizing selection could not be estimated by the OU model. For each analysis, we, therefore, ran several OU models with the *α* parameter fixed at different values, namely 0.1, 0.5, 1 and 10. All models were fitted using the restricted maximum likelihood (REML = T) to allow model comparison. Model significance was then assessed with the ‘Anova’ function of the ‘car’ R package [36]. This ANOVA was carried out on the best model fitted with maximum likelihood (ML) and fixing the phylogenetic parameter to the value obtained with REML. Graphical representation of the regression lines accounted for phylogenetic relatedness by using the regression coefficients computed from the PGLS regressions. Outliers to the regression were defined as species for which the residuals exceeded the threshold of at least 2.5 times that of the median absolute deviation (following [37]). As in Garcia & Ravignani [4], we then constructed 2-by-2 contingency tables (see electronic supplementary material, table S1) pitting how many non-outlier species belong to either VPL clades or to non-VPL clades against how many outliers belong to either VPL clades or to non-VPL clades. This allowed us to test (using Barnard's exact test [38]) whether there is a significant difference between the proportion of outliers in VPL and non-VPL clades, respectively. In addition, we extracted residuals (one value per species) for each of the PGLS best fitted models (four models in total, one for each acoustic parameter-body size regression). Taking the absolute value of residuals, we could then test for differences of magnitude between VPL and non-VPL residuals with the Mann–Whitney *U*-tests (table 1). A similar approach was applied on signed residuals to test for differences in residual distribution between VPL and non-VPL clades (in order to assess if and which of the VPL and non-VPL residuals were above the other and to evaluate whether residuals were either upward or downward outliers relative to the average regression line; table 1). This approach was conducted both on the full dataset, as well as on outliers only (table 1). In all statistical tests, two-tailed statistics were used, and significance levels were set at *α* = 0.05. The same analyses were carried out on the two subsets of data (without Odontocetes, and with only clearly evidenced VPL species).

**Table 1.** For each PGLS regression, comparisons (using the Mann–Whitney *U*-tests) between residuals from VPL and non-VPL clades, either based on the full dataset (*N* = 164 species) or only outliers, and either using the absolute residual values or the signed residual values.

dataset	PGLS regression	MinDF		MaxDF		MeanDF		RangeDF	
		VPL	non-VPL	VPL	non-VPL	VPL	non-VPL	VPL	non-VPL
full dataset	mean signed residuals	0.72	−0.01	0.34	−0.2	0.36	−0.21	0.27	−0.16
	Mann–Whitney *U*-test (VPL/non-VPL)	*W* = 4893	*p* < 0.001	*W* = 5016	*p* < 0.001	*W* = 5171	*p*<0.001	*W* = 4630	*p*<0.001
	mean absolute residuals	0.81	0.35	0.48	0.36	0.48	0.35	0.49	0.39
	Mann–Whitney *U*-test (VPL/non-VPL)	*W* = 4460	*p*<0.001	*W* = 3868	*p* = 0.006	*W* = 3942	*p* = 0.003	*W* = 3841	*p* = 0.008
outliers only	mean signed residuals	1.46	0.31	0.61	−0.94	0.75	−0.92	0.35	−0.67
	Mann–Whitney *U*-test (VPL/non-VPL)	*W* = 193	*p* = 0.002	*W* = 140	*p* < 0.001	*W* = 81	*p* = 0.001	*W* = 198	*p* = 0.007
	mean absolute residuals	1.46	1.18	0.94	1.07	1.01	1.07	0.92	1.1
	Mann–Whitney *U*-test (VPL/non-VPL)	*W* = 181	*p* = 0.009	*W* = 56	*p* = 0.25	*W* = 37	*p* = 0.6	*W* = 85	*p* = 0.11

## 3. Results and discussion

### (a) Fitting regressions: all acoustic features but frequency range are predicted by body size

Model selection indicated that a Brownian model of evolution using Pagel's *λ* was the best evolutionary scenario in the PGLS regression on MinDF, and a Brownian model of evolution using Grafen's *ρ* was the best evolutionary scenario in all other PGLS regressions (MaxDF, MeanDF and RangeDF; see electronic supplementary material, table S2). In line with predictions from acoustic allometry scaling, PGLS regressions were found to be significant for MinDF, MaxDF and MeanDF models, overall demonstrating a negative correlation between body size and acoustic features (MinDF: estimate ± s.e. = −0.37 ± 0.05, *t* = −7.18, *p* < 0.001; MaxDF: estimate ± s.e. = −0.10 ± 0.04, *t* = −2.3, *p* = 0.02; MeanDF: estimate ± s.e. = −0.12 ± 0.04, *t* = −2.73, *p* = 0.007). The regression from the RangeDF model was not significant (estimate ± s.e. = −0.07 ± 0.05, *t* = −1.47, *p* = 0.14), which is in line with the idea that there is no *a priori* expectation to find a negative correlation between body size and frequency range (i.e. that larger-sized species exhibit a lower frequency range than smaller-sized species). The key interest of using this parameter here is tied to the underlying assumption that VPL species may have increased control over their vocal apparatus leading to more advanced frequency modulation, hence a potentially larger frequency range (see §3d for results and discussion on the interaction between frequency range and VPL abilities).

Additional regressions with the two subsets match the results above quite well (see ‘Additional analyses’ in electronic supplementary material). The dataset with no Odontocetes shows significant regressions and negative allometric scaling between body mass and MinDF, MaxDF and MeanDF (but not RangeDF). The dataset where only VPL species (but no VPL clades) are included shows significant regressions and negative allometric scaling between body mass and all acoustic parameters.

### (b) Contingency tables: belonging to a vocal production learning clade associates with being an outlier

**Figure 1.**
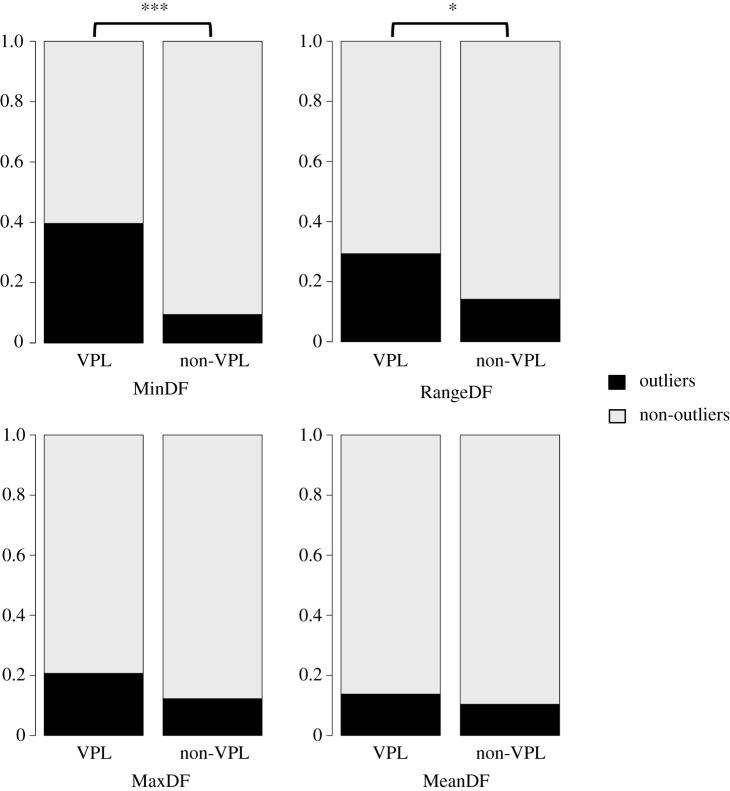
Bar charts representing the proportions of allometric outliers within VPL and non-VPL clades, for each of the four allometric regressions considered in this study. Statistical significance is indicated with asterisks (*p < 0.05, ***p < 0.001). The proportion of allometric outliers is significantly greater in VPL than non-VPL clades when investigating acoustic allometry scaling based on MinDF and RangeDF, but not MaxDF and MeanDF (see electronic supplementary material, table S1).

Similar to some of our previous results, we found a significantly greater proportion of VPL species (when compared with the proportion of non-VPL species) as outliers to the PGLS regressions for MinDF and RangeDF (figure 1; electronic supplementary material, table S1; Barnard's tests: MinDF: *Z* = −4.62, *p* < 0.001; RangeDF: *Z* = −2.34, *p* = 0.02) but not for MaxDF (*Z* = −1.44, *p* = 0.16) and MeanDF (*Z* = −0.65, *p* = 0.58). This suggests that there is an association (despite only for certain acoustic features) between being an outlier to acoustic allometry scaling and having VPL abilities. In addition, the result on RangeDF indicates that an association exists between being an outlier for frequency range and having VPL abilities. This does not contrast with the non-significant result obtained from our PGLS regression on RangeDF; rather, it suggests that despite a lack of association between RangeDF and body mass across species, distributions of RangeDF do differ between VPL and non-VPL species.

Additional tests on contingency tables from the two subsets partly match the results above (see ‘Additional analyses’ in electronic supplementary material). The dataset with no Odontocetes shows a significant association between being a VPL and being an allometric outlier for MaxDF and MeanDF but not for RangeDF and MinDF. Interestingly, if Odontocetes drove the VPL–allometric outlier association found in the main analysis because of echolocation calls, we would expect MaxDF to be a significant predictor in the main analysis and not in the dataset with no Odontoces. As we do not find a significant association between VPL and being an allometric outlier when considering MaxDF in the main analysis, we believe this result further justifies including Odontocetes in our analyses. The dataset where only VPL species in the strict sense (but no VPL clades) are included shows significant associations for RangeDF and MeanDF but not for MaxDF and MinDF. While a clear difference emerges between this (significant MeanDF) and the full dataset (significant MinDF) analyses, RangeDF appears in both cases: there is a greater proportion of VPL species (both when considering VPL species in the strict sense and species in VPL clades) that are allometric outliers for RangeDF, when compared with non-VPL species. The small sample size used for the analyses limited to strictly evidenced VPL species might explain the proportion differences from the main analysis and why a significant association is found for MinDF in one case (the analysis with VPL clades) and MeanDF in the other (the analysis with VPL species only).

### (c) Magnitude and direction of residuals: the regression line as watershed between vocal production learning and non-vocal production learning clades

**Figure 2.**
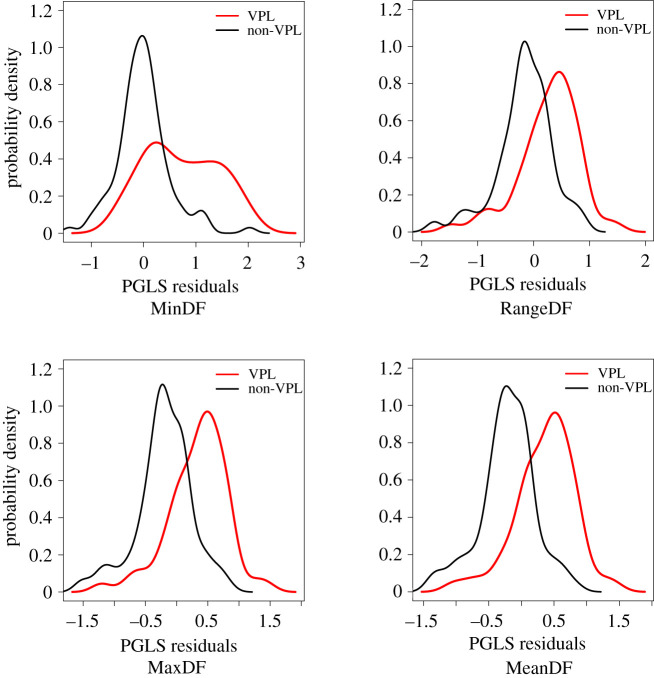
For each PGLS regression, density plots showing the distribution of signed residuals for VPL and non-VPL species.

Investigating the distribution of the residuals for VPL and non-VPL species, we found significant differences in all four regressions (figure 2 and table 1). These results held both when looking at the magnitude of the deviation from acoustic allometry (i.e. based on the absolute residual values in VPL and non-VPL species) as well as the direction of this deviation (i.e. based on the signed residual values in VPL and non-VPLs). More specifically, VPL species showed, on average, a larger deviation from acoustic allometry scaling than non-VPL species, and VPL species were on average above non-VPL species in the acoustic allometry regressions considered (table 1). In addition, the mean signed residual values indicated that VPL species are generally found upward from the average regression line while non-VPL species are on average found downward from the regression line (see the ‘full dataset’ section in table 1: average signed residuals are positive for VPL species and negative for non-VPL species).

**Figure 3.**
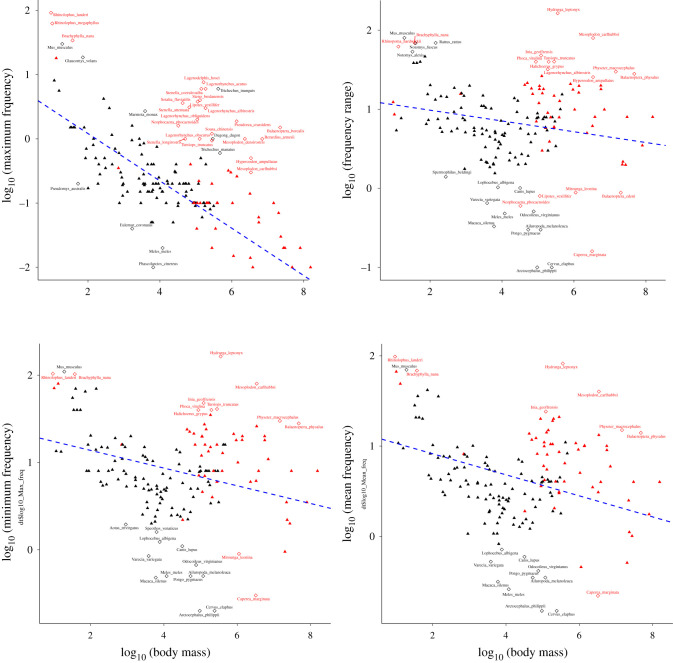
PGLS regressions representing acoustic allometry relationships between acoustic features and body mass (all variables log-transformed). Clockwise, from top-left, MaxDF, RangeDF, MeanDF, and MinDF. VPL species are indicated in red, while non-VPL species are indicated in black. Outliers (see defining criteria in Methods) are indicated by empty diamonds, while non-outliers are indicated by filled triangles. Apart from the regression involving frequency range (top-right panel), all regressions showed that acoustic features are significantly predicted by body mass (see electronic supplementary material, table S2).

Partly similar conclusions were obtained when considering outlier species only (instead of the full dataset): here, VPL outliers showed a larger deviation from acoustic allometry scaling than non-VPL outliers only for MinDF (based on absolute residuals); in addition, as when using the full dataset, VPL species were systematically above non-VPL species in the acoustic allometry regressions considered (based on signed residuals—table 1). Finally, apart from MinDF, for which signed residuals were positive both for VPL and non-VPL outliers, all other regressions showed that VPL outliers were on average found above the regression line (averaged signed residuals are positive for VPL outliers) and non-VPL outliers on average below the regression line (averaged signed residuals were negative for non-VPL outliers—see ‘outliers only’ section in table 1). This is confirmed by visualization of outliers on the PGLS regressions (figure 3): indeed, most VPL outliers are found upward from the regression line in all four regressions. In comparison, most non-VPL outliers are found downward from the regression line (except for MinDF where non-VPL outliers are equally distributed above and below the regression line—figure 3 and table 1). This striking difference suggests that, on average and focusing specifically on outliers to the PGLS regressions, VPL outliers vocalize with higher frequency and larger frequency range vocalizations than expected from standard acoustic allometry scaling, while non-VPL outliers vocalize with lower frequency (except for MinDF: mean signed residuals = 0.31—table 1) and smaller frequency range vocalizations than expected.

Additional tests on the magnitude and direction of residuals from the two subsets partly differ from, though are still consistent with, the results above (see ‘Additional analyses’ in electronic supplementary material). The dataset with no Odontocetes shows (i) significant differences in signed residuals between VPL versus non-VPL species (in other words, VPL species are generally placed significantly higher than non-VPL species relative to the allometric regressions); (ii) mainly no significant differences in absolute residuals between VPL and non-VPLs (except for MaxDF and MeanDF when comparing absolute residuals of species using the full dataset). This suggests that the magnitude of deviation from standard allometric scaling is similar in VPL and non-VPL outlier species, although it can differ when looking at all species instead of outliers only (in which case it shows larger deviations from allometric scaling in VPL than in non-VPL species). The dataset where only VPL species (but no VPL clades) are included shows (i) systematic, significant differences in signed residuals between VPL versus non-VPL species (for all acoustic parameters except MinDF); (ii) no significant differences in absolute residuals between VPL strict and non-VPLs when considering outliers only. When comparing all species, however, we found significant differences in absolute residuals between VPL strict and non-VPL species for all parameters except for MinDF. Overall, these results again suggest larger deviations from allometric scaling in VPL species than in non-VPL (at least when considering the full dataset instead of outliers only) and show that VPL species are found above non-VPL species in the allometric regressions considered in this study.

### (d) How do vocal production learning species influence acoustic allometry regressions?

Finding outliers in a regression is somehow a chicken and egg problem: potential outliers also contribute to defining the regression line which will later qualify them as outliers. In other words, if an association between VPL abilities and deviation from acoustic allometry scaling did exist, it could alter the allometric relationship investigated in this study. Therefore, for comparison purposes, we carried out the same PGLS analyses after removing all VPL species (*N* = 58) from the original dataset (remaining = 106 species). The regressions carried out on this ‘VPL-free’ dataset showed similar results (i.e. significant negative correlations between acoustic measures and body size) for MinDF, MaxDF and MeanDF (see electronic supplementary material, table S3 and figure S1). A notable difference with respect to models fitted using the full dataset appears with RangeDF; here, the VPL-free (as opposed to the full) model also showed a significant negative correlation (electronic supplementary material, table S3). This suggests that adding VPL species to the dataset cancels out an overall RangeDF–body size association, and is likely driven by the fact that vocalizations from VPL species have larger frequency ranges. This is supported both by VPL species vocalizing with larger frequency ranges (based on raw RangeDF values corrected for body size: mean RangeDF ± s.e. in VPL species = 151.16 ± 90.27 Hz g^−1^; mean RangeDF ± s.e. in non-VPL species = 127.4 ± 47.45 Hz g^−1^; Mann–Whitney *U*-test: *W* = 1309, *p* < 0.001) and by significantly higher signed residuals for VPL than non-VPL species (table 1). Similarly, adding VPL species weakens acoustic allometric relationships for MaxDF and MeanDF, which both showed more negative estimates (i.e. steeper slopes) in the VPL-free models. This is likely due to VPL species vocalizing with higher MaxDF and MeanDF than non-VPL species, which is supported both by raw values for MaxDF and MeanDF corrected for body size (MaxDF: mean ± s.e. in VPL species = 464.77 ± 248.3 Hz g^−1^; mean ± s.e. in non-VPL species = 156.91 ± 61.41 Hz g^−1^; Mann–Whitney *U*-test: W = 1353, *p* < 0.001; MeanDF: mean ± s.e. in VPL species = 389.19 ± 220.12 Hz g^−1^; mean ± s.e. in non-VPL species = 93.21 ± 38.19 Hz g^−1^; Mann–Whitney *U*-test: *W* = 1364, *p* < 0.001) and by the significant differences between signed residuals (table 1). Contrary to other models, the VPL-free regression run with MinDF showed a less negative estimate (i.e. a gentler slope), suggesting that adding VPL species to the dataset strengthens the overall MinDF–body mass association. This is likely due to VPL species vocalizing with higher MinDF than non-VPL species, as indicated by raw MinDF values corrected for body size (mean MinDF ± s.e. in VPL species = 313.61 ± 198.32 Hz g^−1^; mean MinDF ± s.e. in non-VPL species = 29.51 ± 16.46 Hz g^−1^; Mann–Whitney *U*-test: *W* = 1277, *p* < 0.001) and by the significant difference between signed residuals (table 1). Note that in the above, raw acoustic values are actually expressed in Hz g^−1^ (Hertz per gram) in order to control for potential systematic differences in body size between VPL and non-VPL species.

### (e) How are vocal production learning and non-vocal production learning outliers positioned along the acoustic allometry regressions?

We investigated which species may have a potential for possessing VPL abilities by looking at which species were found to be outliers (and if so whether upward or downward) from acoustic allometry scaling for at least one acoustic parameter in either of the PGLS regressions on the full dataset (see tables 2 and 3). Table 2 has a particularly distinctive feature: identified outlier cases mainly consist of upward outliers (52/60), suggesting that outliers in VPL clades produce higher call frequencies than expected. While cetaceans and bats are often featured in the list of outlier species, this also includes several pinnipeds. Table 2, therefore, overall indicates that the outlier status is not fully explained by high-frequency echolocation signals. By contrast, table 3, which includes all non-VPL outliers from the PGLS regressions on the full dataset, shows that the majority of outliers are downward outliers (37/49), suggesting that outliers in non-VPL clades produce lower call frequencies than expected. In addition to the results and insights described above, running VPL-free PGLS regressions (i.e. without clades presently thought to comprise VPL species) allowed identifying further allometric outliers and, therefore, a scan for further species with potential VPL abilities (see electronic supplementary material, table S4).

**Table 2.** List of VPL species found as outliers to allometry scaling for each of the four models using the full dataset. The direction of the deviation from acoustic allometry scaling is indicated either as U (denotes an upward outlier, i.e. one above the regression line) or D (denotes a downward outlier, i.e. one below the regression line). *N* = 33 species.

binomial name	common name	MaxDF	MeanDF	MinDF	RangeDF	category
*Balaenoptera borealis*	sei whale			U		1
*Balaenoptera edeni*	Bryde's whale				D	3
*Balaenoptera physalus*	fin whale	U	U		U	1
*Berardius arnuxii*	Arnoux's beaked whale			U		
*Brachyphylla nana*	Cuban fruit-eating bat	U	U	U	U	1
*Caperea marginata*	pygmy right whale	D	D		D	3
*Halichoerus grypus*	grey seal	U			U	1
*Hydrurga leptonyx*	leopard seal	U	U		U	1
*Hyperoodon ampullatus*	northern bottlenose whale			U	U	1
*Inia geoffrensis*	Amazon river dolphin	U	U		U	1
*Lagenodelphis hosei*	Fraser's dolphin			U		1
*Lagenorhynchus acutus*	Atlantic white-sided dolphin			U		1
*Lagenorhynchus albirostris*	white-beaked dolphin			U		1
*Lagenorhynchus obliquidens*	Pacific white-sided dolphin			U		1
*Lagenorhynchus obscurus*	dusky dolphin			U		1
*Lipotes vexillifer*	Baiji dolphin			U	D	5
*Mesoplodon carlhubbsi*	Hubbs' beaked whale	U	U	U	U	1
*Mesoplodon densirostris*	Blainvolle's beaked whale			U		1
*Mirounga leonina*	southern elephant seal	D			D	3
*Neophocaena phocaenoides*	finless porpoise			U	D	5
*Phoca vitulina*	harbour seal	U			U	1
*Physeter macrocephalus*	sperm whale	U	U		U	1
*Pseudorca crassidens*	false killer whale			U		1
*Rhinolophus landeri*	Lander's horseshoe bat	U	U	U		1
*Rhinolophus megaphyllus*	smaller horseshoe bat			U		1
*Rhinopoma hardwickii*	lesser mouse-tailed bat				U	1
*Sotalia fluviatilis*	tucuxi dolphin			U		1
*Sousa chinensis*	Indo-Pacific humpbacked dolphin			U		1
*Stenella attenuata*	pantropical spotted dolphin			U		1
*Stenella coeruleoalba*	striped dolphin			U		1
*Stenella longirostris*	spinner dolphin			U		1
*Steno bredanensis*	rough-toothed dolphin			U		1
*Tursiops truncatus*	bottlenose dolphin	U		U	U	1

**Table 3.** List of non-VPL species found as outliers to allometry scaling for each of the four models using the full dataset (see electronic supplementary material, table S4 for a table specifically displaying similar model outputs based on the ‘VPL-free’ models instead of the full dataset). The direction of the deviation from acoustic allometry scaling is indicated either as U (denotes an upward outlier, i.e. one above the regression line) or D (denotes a downward outlier, i.e. one below the regression line). *N* = 25 species.

binomial name	common name	MaxDF	MeanDF	MinDF	RangeDF	category
*Ailuropoda melanoleuca*	giant panda	D	D			2
*Aotus trivirgatus*	three-striped night monkey	D				2
*Arctocephalus philippii*	Juan Fernández fur seal	D	D		D	2
*Canis lupus*	wolf	D	D		D	2
*Cervus elaphus*	red deer	D	D		D	2
*Dugong dugon*	dugong			U		4
*Eulemur coronatus*	crowned lemur			D		2
*Glaucomys volans*	southern flying squirrel			U		4
*Lophocebus albigena*	grey-cheeked mangabey	D	D		D	2
*Macaca silenus*	lion-tailed macaque	D	D		D	2
*Marmota monax*	groundhog			U		4
*Meles meles*	European badger	D	D	D	D	2
*Mus musculus*	house mouse	U	U	U	U	4
*Notomys alexis*	spinifex hopping mouse				U	4
*Notomys fuscus*	dusky hopping mouse				U	4
*Odocoileus virginianus*	white-tailed deer	D	D		D	2
*Phascolarctos cinereus*	koala			D		2
*Pongo pygmaeus*	Bornean orangutan	D	D		D	2
*Pseudomys australis*	plains rat			D		2
*Rattus rattus*	black rat				U	4
*Speothos venaticus*	bush dog	D				2
*Spermophilus beldingi*	Belding's ground squirrel				D	2
*Trichechus inunguis*	Amazonian manatee			U		4
*Trichechus manatus*	West Indian manatee			U		4
*Varecia variegata*	black-and-white ruffed lemur	D	D		D	2

### (f) A brief overview of outlier categories identified from our phylogenetic generalized least square regressions

From tables 2 and 3 summarizing outlier cases, five main categories emerge across species. The first two categories contain a large number of species and, combined, most outliers. They conform to the results described above, with VPL species being mainly upward outliers (category 1; *N* = 28/33) and non-VPL species being mainly downward outliers (category 2; *N* = 16/25). Anatomical adaptations could be a parsimonious explanation for non-VPL species being downward outliers (as seen e.g. with koalas and red deer—table 3). In parallel, one could hypothesize that advanced vocal flexibility explains the results for VPL species, as this would also allow them to exploit the high-frequency acoustic space that is typically not targeted by mere anatomical adaptations. In contrast, categories 3, 4 and 5 challenge the hypothesis that vocal learners are upward outliers but only contain 14 species overall.

Category 3 features species which belong to VPL clades but are consistent downward outliers. This is the case for three species, namely *Balaenoptera edeni*, *Caperea marginata* and *Mirounga leonina*. These species could either be exceptions to our hypothesis, not full-fledged vocal learners or may combine VPL and anatomical adaptations. For instance, in southern elephant seals (*Mirounga leonina)*, a potential capacity for VPL [39] could coexist with anatomical adaptations (their proboscis) which may both be driven by strong sexual selection and push sound frequencies downwards [40].

Category 4 includes species which are not traditionally recognized as belonging to VPL clades, but are nonetheless upward outliers in our regressions. These are: *Dugong dugon*, *Glaucomys volans*, *Marmota monax*, *Mus musculus*, *Notomys alexis*, *Notomys fuscus*, *Rattus rattus*, *Trichechus inunguis* and *Trichechus manatus*. It may be that the VPL capacities of these species have not been fully understood, and our hypothesis points towards them as potential candidates for VPL.

The last category (category 5) includes two VPL species (*Lipotes vexillifer* and *Neophocaena phocaenoides*) which come out as both upward (MinDF) and downward (RangeDF) outliers. Thus, these species seem to have both a higher minimum frequency and a smaller frequency range than that predicted for their body size. While these species probably do not have much value to test our framework, they may be methodologically meaningful: they show that a few species (<2% overall) can display peculiar combinations of which parameters are outliers in which directions, but most species considered here do not. In addition, category 5 provides evidence that all four acoustic parameters are not necessarily redundant.

Pushing our examination further, we found supporting evidence for the hypothesis that VPL species may use a high-frequency acoustic space. VPL species are typically upward outliers for MaxDF (10/12; table 2) and MinDF (23/23; table 2). In addition, table 2 shows that most of the VPL species found to be upward outliers for MaxDF are also upward outliers for RangeDF (*N* = 8/10). For non-VPL clades, as described above, we found a majority (37/49; table 3) of downward outliers. This was the case for MaxDF (downward outliers = 12/13), MeanDF (downward outliers = 10/11 and RangeDF (downward outliers = 11/15) but not for MinDF (downward outliers = 4/10). The results on MaxDF and RangeDF indicate that non-VPL species may not fully exploit the higher frequency range of their vocal production, thereby limiting both their MaxDF and their RangeDF. However, the result on MinDF starkly contrasts with our original hypothesis [4] since we predicted non-VPL species to be typically downward outliers for MinDF, in line with anatomical adaptations and their associated allometric deviations resulting from selection for size exaggeration. A possible explanation for this surprising result would be that non-VPL upward outliers are, in fact, VPL species, overall highlighting the potential of this study when it comes to identifying candidate VPL species. Looking in particular at the MinDF column in table 3, future research will either support or refute these hypotheses by investigating (i) whether anatomical adaptations can be found in downward non-VPL outliers (namely *Eulemur coronatus*, *Meles meles* and *Pseudomys australis*—note that such evidence has already been demonstrated for the fourth species, *Phascolarctos cinereus* [17]) and (ii) whether VPL abilities can be found in upward non-VPL outliers (*Dugong dugon*, *Glaucomys volans*, *Marmota monax*, *Mus musculus*, *Trichechus inunguis* and *Trichechus manatus—*note that, among those species, *Mus musculus* represents a debated case of vocal learning abilities [41,42] and Sirenia (*Dugong dugon*, *Trichechus inunguis* and *Trichechus manatus*) are closely related to elephants [43], in turn an established vocal learning clade [25]). Finally, in most cases (45/51), species found to be outliers in the MeanDF and RangeDF are also outliers in either the MaxDF or the MinDF regressions. This suggests that results should be interpreted carefully because, *a priori*, the four acoustic parameters investigated here have only 2 degrees of freedom. At the same time, the four measures are not completely redundant, in the sense that two are not enough to explain all of them. Empirical examples of this come from the 6/51 remaining species: *Balaenoptera edeni* and *Spermophilus beldingi*, which appear as downward outliers for RangeDF only, and *Notomys alexis*, *Notomys fuscus*, *Rattus rattus* and *Rhinopoma hardwickii* which appear as upward outliers for RangeDF only. More in general, the columns in tables 2 and 3 are not linear combinations of each other (i.e. knowing any two columns does not provide a good guess of the other two), again stressing the value of using four acoustic variables instead of two.

Similar analyses carried out on a VPL-free dataset provide the same categories as table 3 (i.e. categories 2, 4 and 5—see electronic supplementary material, table S4). These regressions offer additional leads to identifying species with either potential anatomical adaptations (the downward outliers) or potential VPL abilities (the upward outliers). Altogether, the observations collected in this study on allometric outliers (tables 2 and 3; electronic supplementary material, table S4) highlight possible convergence towards deviation from allometry scaling. Although our results do not demonstrate VPL abilities *per se*, they advocate the utility of conducting comparative research on deviations from acoustic allometry scaling, and help inform about which species could represent particularly well-suited candidates when testing for VPL abilities across mammals.

Our additional analyses carried out with no Odontocetes and with VPL species only also fall in line with the above categorization and general results: in both analyses, VPL outliers are mostly found upward from the allometric regression while non-VPL outliers are found both upward and downward from it (see electronic supplementary material, tables S8, S9, S13 and S14 for details). Note that the species found as outliers also greatly overlap in all analyses, despite slight differences inherent to the use of different datasets.

### (g) Fit of the data with alternative explanations for the evolution of vocal production learning

Vocalizing at lower frequencies should minimize muscular contractions, both from laryngeal muscles controlling vocal fold tension through elongation, and respiratory muscles controlling the subglottal pressure applied to the air expelled through the trachea and vocal apparatus [20,44–46]). It may thus be physiologically easier to vocalize closer to the lowest achievable frequency than to the highest achievable frequency, because the former is achieved with a relaxed vocal fold state (i.e. low tension on the vocal folds) and limited amount of air expelled through the glottis (i.e. reduced subglottal pressure), while the latter is achieved through strenuous conditions (strong tension applied on the vocal folds and/or increased subglottal pressure) [46]. Because VPL species are assumed to display a higher degree of vocal control [1,5,47], such control may provide them with an advantage to reach higher frequencies more easily than non-VPL species. This could overall explain why most VPL species (45/58) are found above, and not below, the average regression line for RangeDF.

Nowicki & Searcy [3] proposed five hypotheses on potential selective benefits and mechanisms which, in birds, could lead to the evolution of vocal learning. These are: (1) the vocal dialect hypothesis (where local genetic adaptation is achieved through the influence of local dialects on assortative mating); (2) the sexual selection hypothesis (where increased mating success is driven by the effect of repertoire expansion on mate choice); (3) the information sharing hypothesis (where enhanced fitness of kin is achieved by an improved information sharing due to repertoire expansion); (4) the environmental adaptation hypothesis (where more effective signalling is based on an improved sound transmission allowed by fitting signals to the environment); (5) the individual recognition hypothesis (where enhanced social interactions are achieved through a selection for vocal distinctiveness that improves individual recognition). We do not advocate for any of these hypotheses in particular, as we believe that all can in principle fit the patterns observed here in the data for VPL clades. In particular: (1) developing local dialects for assortative mating may lead to an exploration of the acoustic space and hence to reaching more extreme frequencies in at least some of the species' subpopulations; (2,3) repertoire expansion for mate choice or information sharing suggests that the expanded repertoire be at least as large in size as the initial repertoire (thus potentially pushing the frequency range extrema). Note, however, that this hypothesis may predict that VPL species have both lower minimum frequency and higher maximum frequency, which we do not see in the data presented here (in particular since VPL species also often have vocalizations with higher minimum frequencies than expected—table 2); (4) fitting signals to the environment for the purpose of improved transmission may also push the repertoire towards an extreme (however, note that in this case, narrowing of the repertoire is an alternative possible result of selection for improved transmission, and would suggest that this mechanism does not match well with the data observed here); (5) within a population, distinctiveness for individual recognition may also push the individual acoustic boundaries once the core central frequencies are saturated by other conspecifics. Overall, the above emphasizes the fact that although different paths could lead to the evolution of VPL, the end result that consists in deviating from acoustic allometry scaling may represent a shared acoustic trait across most VPL species.

While our approach mainly consisted in identifying deviations from allometric scaling in VPL clades, several scenarios may provide causal explanations to our results. First, as mentioned in our original work [4], several selective pressures may drive allometry-cheating strategies among mammals, regardless of VPL abilities. Examples of such pressures include temperature and metabolic conditions [48,49], particular foraging and orientation strategies involving the production of echolocation signals (e.g. in bats and dolphins) [50], anatomical adaptation to an aquatic lifestyle (such as phonic lips in Odontocetes) [51], as well as environmental noise (whether biotic [52] or abiotic [53]). Note, in particular, that most vocal learning mammals are not typical terrestrial quadrupeds, but rather aquatic (cetaceans), semi-aquatic (pinnipeds) or terrestrial flying animals (bats). Therefore, our and any cross-species analysis needs to consider these species’ adaptation to a highly three-dimensional lifestyle: this factor, rather than VPL, could be the main driver of allometric deviations. Likewise, the fact that several vocal learners also echolocate introduces an additional bias. Unfortunately, until more species of terrestrial vocal learners are discovered, it will be difficult to disentangle these factors. Second, selective pressures may also have favoured the emergence of vocal learning abilities without necessarily affecting acoustic allometry relationships. For instance, Fischer *et al*. [11] recently demonstrated that vocal convergence in Guinea baboons was dependent on the amount of social interactions between individuals, therefore showing that some implicit forms of vocal learning can likely originate from social factors, and that allometry scaling need not be affected for a species to exhibit vocal learning abilities. In addition to confirming other vocal convergence results in this clade [21], Fischer *et al*.'s study also reaffirms the need to consider vocal learning as a multidimensional space transcending the limitation of vocal learning to ‘vocal production learning’, even when the latter is thought of as a continuum [11,12]. In summary, although our current analyses provide novel insights into the association between VPL and allometric scaling, they do not provide strong support for sexual selection being the only mechanism responsible for this association, as we instead originally hypothesized [4].

## 4. Limitations and conclusion

As described in Methods, using dominant frequencies represents a compromise between the choice for either source-related or filter-related parameters. While this analysis equally applies to all species (regardless of whether they are VPL or non-VPL species), we invite future empirical studies to further test our hypotheses by collecting and analysing data featuring source-only (e.g. the fundamental frequency *f_0_* and its harmonics) and filter-only acoustic parameters (e.g. formant frequencies and their spacing) to disentangle the effect of potential selection pressures on these two components of animal sound production.

In our previous work, we found that VPL status was significantly associated with being an outlier for MinDF but not MaxDF. We originally interpreted this as MinDF being more reliable than MaxDF to investigate acoustic allometric relationships, since MinDF would generally coincide with *f*_0_ in tonal calls and with the first formant in harsh vocalizations. Our data still offer some support for our original interpretation, at least when investigating acoustic allometry in the context of size exaggeration. Our current results however point at the validity and importance of also considering MaxDF while looking at the interaction between acoustic allometry and vocal modulation, because enhanced vocal flexibility may push the upper frequency boundary and lead to an increased frequency range.

Overall, our study proposes that some non-VPL species are generally found as downward outliers to acoustic allometry regressions over body size, most likely because of evolutionary pressures leading to anatomical adaptations for size exaggeration. To the contrary, VPL species are generally found as upward outliers, which we suggest could highlight a shift in evolutionary selection pressures for an increased vocal modulation, thereby departing from size exaggeration processes. If this connection holds, direction and amount of deviation from allometric scaling may be used as a diagnostic tool for untapped vocal learners. In this sense, this work may offer a novel tool towards the identification of new candidate VPL species.

## Endnotes

1 Note that the word ‘predictor’ is used here in its statistical sense, not to denote causality.

2 Conversely, in some cases, deviation from allometry can arise from mechanisms and adaptations which are neither honest nor dishonest *per se*, e.g. echolocation calls and their associated partitioning of sonar frequency bands [15].

## Data accessibility

Data used for this study are available as an electronic supplementary material raw data file.
