# Correction to ‘A cross-species framework to identify vocal learning abilities in mammals’

**DOI:** 10.1098/rstb.2021.0489

**Published:** 2022-01-31

**Authors:** Andrea Ravignani, Maxime Garcia

*Phil. Trans. R. Soc. B* **377**, 20200394. (Published online 15 November 2021). (doi:10.1098/rstb.2020.0394)

The originally published version of this paper showed an error in figure 3. The *Y*-axis titles in the first column were reversed (upper left panel incorrectly showed ‘log10(maximum frequency)’ and the lower left panel showed ‘log10 (minimum frequency)’). The correct figure is shown below. This has also been corrected on the publisher's website.

**Figure 3.**
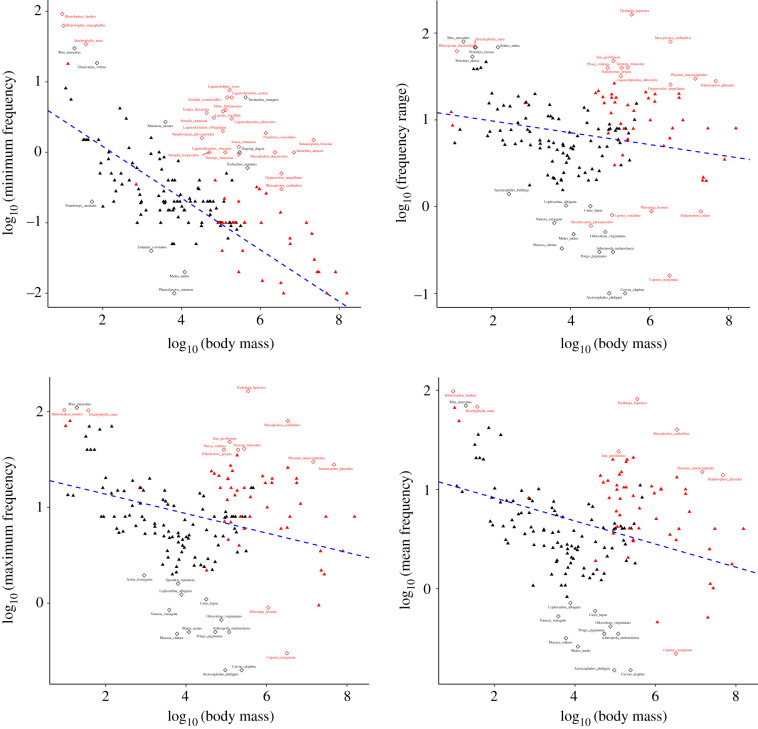
PGLS regressions representing acoustic allometry relationships between acoustic features and body mass (all variables log-transformed). Clockwise, from top-left, MaxDF, RangeDF, MeanDF, and MinDF. VPL species are indicated in red, while non-VPL species are indicated in black. Outliers (see defining criteria in Methods) are indicated by empty diamonds, while non-outliers are indicated by filled triangles. Apart from the regression involving frequency range (top-right panel), all regressions showed that acoustic features are significantly predicted by body mass (see electronic supplementary material, table S2).

